# Spatial variation and determinants of inappropriate complementary feeding practice and its effect on the undernutrition of infants and young children aged 6 to 23 months in Ethiopia by using the Ethiopian Mini-demographic and health survey, 2019: spatial and multilevel analysis

**DOI:** 10.3389/fpubh.2023.1158397

**Published:** 2023-10-30

**Authors:** Nebiyu Mekonnen Derseh, Daniel Alayu Shewaye, Muluken Chanie Agimas, Meron Asmamaw Alemayehu, Fantu Mamo Aragaw

**Affiliations:** Department of Epidemiology and Biostatistics, Institute of Public Health, College of Medicine and Health Sciences, University of Gondar, Gondar, Ethiopia

**Keywords:** spatial variation, inappropriate complementary feeding practice, infants and young children, undernutrition, Ethiopia, spatial and multilevel analysis

## Abstract

**Background:**

Inappropriate complementary feeding practices (IACFPs) are major public health issues in Ethiopia, which usually result in stunting and becoming an intergenerational cycle. However, the spatial patterns and determinants of IACFP and its effect on undernutrition are not well understood in Ethiopia. Therefore, the aim of this study was to explore the spatial patterns and determinants of IACFPs and their effects on the undernutrition of infants and young children (IYC) in Ethiopia.

**Methods:**

This was a nationwide community-based survey study among 1,463 mothers of IYC aged 6–23 months in Ethiopia. The global spatial autocorrelation was assessed using the global Moran’s-*I* to evaluate the spatial clustering of IACFPs. Significant clusters with high and low rates of IACFPs were explored. A multilevel mixed-effect logistic regression with cluster-level random effects was fitted to identify determinants of IACFPs with an AOR and 95% CI.

**Results:**

The prevalence of IACFPs was 90.22%. The spatial pattern of IACFP in Ethiopia was clustered across regions (Global Moran’s I = 0.63, Z-score = 12.77, value of *p* ≤0.001). Clusters with a high rate of IACFP were detected in southern, northwest, and eastern Ethiopia. Individual and community-level variables accounted for 63% of IACFP variation. Mothers with no education were 3.97 times (AOR = 3.97; 95% CI: 1.64–9.60) more likely to have IACFPs than those with higher education. The poorest HHs had 4.80 times the odds of having IACFPs as the richest HHs (AOR = 4.80, 95% CI: 1.23–18.71). The odds of having IACFPs were 2.18 times (AOR = 2.18; 95% CI: 1.28–3.72) higher among babies with no postnatal checkup. Non-breastfed IYC were 2.8 times (AOR = 2.80; 95% CI: 1.29–6.10) more likely to have IACFP when compared with breastfed ones. IYC with the inadequate introduction of solid, semi-solid, and soft foods, inadequate minimum dietary diversity, and inadequate minimum acceptable diet were more likely to have wasting, underweight, and stunting.

**Conclusion:**

This study showed the prevalence of IACFPs was very high, which had an effect on undernutrition and showed spatial variation in Ethiopia. Therefore, the government of Ethiopia and stakeholders should focus on women with no education and the poorest HHs, encourage PNC checkups, and encourage breastfeeding in the hotspot areas to minimize IACFPs in Ethiopia.

## Background

Complementary feeding (*CF*) is defined as the use of age-appropriate, adequate, and safe solid or semi-solid foods in addition to breast milk for children aged 6 to 23 months (1). Appropriate feeding practice during infancy and early childhood is very important for optimal growth, health, and development (2). The World Health Organization (WHO) recommends that the complementary feeding practice needs to be starting a timely, given in safely, and appropriately with adequate amounts, frequency, and consistency with a variety of foods to fulfill the nutritional requirements in addition to breastfeeding (3). However, the above recommendation is usually overlooked, especially in developing countries.

Inappropriate complementary feeding practices (IACFPs) are one of the main public health issues in the world. Globally, 35.5, 47.8, and 70.6% of IYC were not receiving solid, semisolid, or soft foods (ISSSF), minimum meal frequency (MMF), and minimum diversity (MDD), respectively, which are indicators of appropriate complementary feeding (4). This was the main concern in low-and middle-income countries (LMICs) (5). A study among 80 LMICs showed that only one in four children met the MDD threshold and one in two met the recommended MMF per day (6). A study from Bangladesh showed that 46, 48, and 28% of IYC met MDD, MMF, and the minimum acceptable diet (MAD), respectively (7).

The prevalence of inadequate MAD in Sub-Saharan Africa (SSA) and Eastern Africa (EA) was very high (90% (8) and 76.53% (9), respectively). Similarly, the prevalence of inadequate MDD in the EA was more than three-fourths (76.53%) (10). Likewise, the magnitude of MMF in Western and Central Africa, Eastern and Southern Africa, and Southern Africa was less than 50% and the prevalence of MDD in the above areas was less than 25% (4).

In Ethiopia, the magnitude of inadequate MDD (86%), MMF (45%), and MAD (89%) (11) were very high compared with other African countries, which would result in a high prevalence of IACFP.

Inappropriate complementary feeding practices among IYC usually lead to immediate and long-term health-related complications. Among the immediate effects, many young children might develop acute and chronic malnutrition like wasting, undernutrition, and stunting (12). Moreover, they could have deficiencies in essential micronutrients like vitamin A, vitamin D, and iron (13, 14). As a result, undernourished infants and young children could have low immunity and are easily affected by fatal infectious diseases such as diarrhea, pneumonia, TB, and others. Severe acute malnutrition accounts for more than 41 percent of deaths among infants and young children aged 6 to 24 months each year in developing countries (2). On the other hand, chronic malnutrition in the first two years could be associated with long-term stunting and health-related impacts. Inappropriate complementary feeding practices during the first 2 years usually result in stunting, which later affects the reproductive capacity in women, and this may lead to complicated deliveries and low-birth-weight (LBW) newborns. Without intervention, this becomes a recycling issue within the generation (2). Moreover, stunting before the age of 2 years leads to poor cognitive and academic achievements in later childhood and adolescence period which result in significant educational and economic impacts at the individual, household, and community levels (15).

There were different factors associated with IACFPs in LMICs, including Ethiopia. Mothers with no education, low HH wealth status, no ANC visit, cultural beliefs, the absence of a post-natal check-up, being 6 to 11 months old, and being a non-breastfeeding child were factors associated with inappropriate complementary feeding, as reported by various scholars (10, 16–19).

The government of Ethiopia adopted different strategies to scale up IYCFPs, including promoting optimal breastfeeding by giving training to low-and middle-level healthcare providers that routinely counsel mothers of infants and improving agricultural irrigation and crop production for food security and diversity. However, there were no remarkable changes in the trends of appropriate complementary feeding practices in Ethiopia (11, 20). There were regional and urban–rural variations in *CF* indicators in Ethiopia. According to the 2019 EMDHS, the proportions of MMF and MDD were reported as the lowest in Somali (34 and 1%) and the highest in Addis Ababa (82 and 29%), respectively, and the proportion of MAD in Addis Ababa was 28%, whereas, in Somali, Afar, and Amhara regions, they were 1, 4, and 6% (11). Exploring the spatial distribution of IACFP and identifying its determinants, along with understanding its effect on a child’s undernutrition, are very important in order to intervene in this problem. However, the spatial distribution of IACFP and its effect on undernutrition is not well known nationally. Spatial analysis is used to detect hotspots (high prevalence of IACFPs in certain areas), predict IACFPs in unobserved areas, and explore high and low rates of IACFPs in certain areas, thereby allowing concerned bodies to design interventions accordingly.

Although there was a study of appropriate complementary feeding practices in Ethiopia (21), it did not show the spatial variation of IACFPs at the regional level or the association of IACFPs with the nutritional status of IYC aged 6–23 months, and some important variables were not assessed,which are essential for designing an effective intervention. Therefore, this study aimed to explore the spatial variation and determinants of IACFPS and its effect on the undernutrition of infants and young children aged 6 to 23 months in Ethiopia.

## Methods

### The study settings

This was nationwide a survey study that was conducted regionally in urban and rural areas of Ethiopia from March 21, 2019, to June 28, 2019, as part of the Ethiopian Mini-Demographic and Health Surveys (EMDHS) 2019, which was the second EMDHS implemented in Ethiopia. The first EMDHS was conducted in 2014 (11).

Ethiopia is a land-locked country in the Horn of Africa and lies between the latitudes of 3° and 15° North and the longitudes of 33° and 48° East. It has a total area of 1,100,000 km^2^. There are 11 ethnically and politically autonomous regional states and two administrative cities in Ethiopia. The regions are divided into 68 zones, which are further subdivided into 817 districts, which are then subdivided into about 16,253 kebeles (the lowest locally administered units) (22). Ethiopia is a country with great geographical diversity; its topographic features range from the highest peak at Ras Dejen, which is 4,550 meters above sea level, to the lowest at the Afar Depression, 110 meters below sea level. The climatic condition of the country varies with the topography, and the temperature is as high as 47°C in the Afar depression and as low as 10°C in the highlands. Ethiopian basic economic source is agriculture, which is the backbone of the national economy (23). Ethiopian population growth is rapid (53.5 million in the 1994 census, which has since increased to 114,963,588 with a fertility rate of 4.3 in 2020″ (24).

### Study design and period

A nationwide community-based survey study was conducted with a nationally representative sample of IYC aged 6 to 23 months in Ethiopia from March to June 2019.

### Population and eligibility criteria

The source population for this study was all infants and young children (IYC) in Ethiopia aged 6 to 23 months at the time of the survey, while the study population was all IYC in Ethiopia aged 6 to 23 months who were in the selected EAs and included in the analysis.

### Data source, sample size, and sampling techniques

The data source for this study was the DHS program database, which was accessed through the www.measuredhs.com website after presenting the research domain and objectives. We used a total sample size of 1,463 IYC for this study. Sampling weight was done to maintain representativeness due to the non-proportional allocation of the sample size in different regions and their urban and rural variations, as well as the possible differences in response rates (11). In EMDHS 2019, each region was stratified into urban and rural areas, which were grouped into 21 sampling strata, then the sample was selected in two stages. In the first stage, stratified samples of census enumeration areas (EAs) in urban and rural areas were selected with complete household (HH) listings using systematic probability sampling based on the sampling frame of all census EAs created for the 2019 Ethiopian Population and Housing Census (EPHC) that was conducted by the Central Statistical Agency (CSA). In the second stage, households (HH) were selected using the same probability systematic sampling in the selected EAs. In each selected HH, reproductive-age women with IYC were interviewed with an individual questionnaire (11).

### Study variables

The dependent variable in this study was inappropriate complementary feeding practices (IACFPs). The complementary feeding practice in the i^th^ group of mothers with IYC in the j^th^ cluster (yij) was dichotomized as yij = 1, for those who had IACFPs, whereas yij = 0, for those who had appropriate complementary feeding practices (APCFP).

The outcome variable (IACFP) in this study was measured by the following three WHO composite complementary feeding indicators: 2021 for IYC (25).

Introduction of solid, semi-solid, or soft foods (ISSSF) 6–8 months: percentage of infants 6–8 months of age who consumed ISSSF during the previous day (25).

Minimum dietary diversity (MDD) for 6–23 months: percentage of children 6–23 months of age who consumed foods and beverages from at least five out of eight defined food groups during the previous day (25). A list of eight food groups was: breast milk; grains, roots, and tubers; legumes and nuts; dairy products (milk, yogurt, and cheese); flesh foods (meat, fish, poultry, and liver or organ meat); eggs; vitamin-A-rich fruits and vegetables; and other fruits and vegetables (25).

Minimum meal frequency for 6–23 months: percentage of children 6–23 months of age that consumed ISSSF (but also including milk feeds for non-breastfed children), the minimum number of times or more during the previous day. The minimum number of times was defined as two feedings of ISSSF for breastfed infants aged 6–8 months, three feedings of ISSSF for breastfed children aged 9–23 months, and four feedings of ISSSF or milk feeds for non-breastfed children aged 6–23 months, whereby at least one of the four feeds must be ISSSF (25).

Complementary feeding practices: When infant and young child feeding practices have met three of the above indicators, we consider them “appropriate,” while if one of the three indicators is not met, it is called “inappropriate” (25).

The minimum milk feeding frequency for non-breastfed children (NBFC) aged 6–23 months was defined as the percentage of NBFC aged 6–23 months who consumed at least two milk feeds during the previous day (25).

The minimum acceptable diet for 6–23 months was defined as the percentage of children 6–23 months of age who consumed a minimum acceptable diet during the previous day (25).

Wasting is defined as a weight-for-height z-score that is less than minus 2 (−2.0) SD below the mean on the WHO Child Growth Standards.

Underweight is a weight-for-age z-score below minus 2 (−2.0) SD below the mean on the WHO Child Growth Standards.

Stunting is defined as a height-for-age z-score that is less than minus 2 (−2.0) SD below the mean on the WHO Child Growth Standards.

### Independent variables

Independent variables of IACFPs were extracted according to the literature reviews (18, 26, 27). Individual-level and community-level factors were considered to be determinants of IACFP in Ethiopia. Sociodemographic, socioeconomic, obstetric-related, and child-related factors were included under individual-level factors. Sociodemographic factors included mothers’ educational status, religion, number of living children plus current pregnancy, the number of under-five children, and marital status. Socioeconomic characteristics included a wealth index and HH owns a radio. The wealth index was a composite measure of a household’s cumulative living standard that was divided into 5 quantiles, which were derived by using principal component analysis (22). Among obstetric-related factors, contraceptive use, birth order number, ANC visit, place of delivery, and a post-natal checkup within two months were included. Infants and young children-related factors included the child’s age, breastfeeding status, and VA-1 supplementation at 6 months. Community-level factors included region and place of residence.

### Data collection and tools

The EMDHS data were collected through face-to-face interviews using questionnaires at the individual and household levels. During the data collection period, mothers of IYC aged 6–23 months were asked to give important socio-demographic, socio-economic status, obstetric, and child-related characteristics that were associated with IACFP in Ethiopia (11).

### Data management and analysis

Data were extracted, cleaned, recoded, and labeled for further analysis using STATA 14 and Microsoft Excel. Before conducting the analysis, sampling weights for each variable were calculated to account for the strata’s unequal probability of selection.

#### Spatial analysis

The global spatial autocorrelation was performed using the global Moran’s-*I* to evaluate the spatial variation of inappropriate complementary feeding practices (IACFP) using ArcGIS Version 10.8. Statistically significant positive Moran’s I value indicates a geographical clustering for the IACFP, while statistically significant negative Moran’s Index shows dispersion, and if the value is zero, it shows the random distribution.

The Getis-Ord Local Spatial Statistics Tool was used to identify statistically significant hotspots and cold spots. “Hotspot” refers to the occurrence of a high prevalence of IACFP that clustered together on the map, whereas “cold spot” refers to the occurrence of a low prevalence of IACFP that clustered together on the map.

The ordinary Kriging interpolation method was applied to predict a high prevalence of IACFP in unobserved enumeration areas in Ethiopia.

We explored spatial scan statistics using the Bernoulli probability model to detect local clusters of statistically significant high rates and low rates of IACFPs using SaTScan 10.1. A cluster is statistically significant when its log-likelihood ratio (LLR) is greater than the standard Monte Carlo critical value at a value of p less than 0.05. The maximum likelihood ratio test statistic showed the most primary cluster relative to the global distribution of maximum values. The primary and next further significant clusters were identified, the LLR was assigned, and the value of p was obtained through Monte Carlo hypothesis testing with 999 Monte Carlo replicates.

#### Multilevel analysis

A mixed-effect binary logistic regression model was fitted to identify the possible factors associated with IACFP among mothers of IYC in Ethiopia using STATA 14. We performed a multilevel model because EMDHS data were hierarchical and nested within EAs (11). Therefore, a two-level model was fitted by considering secondary sampling units as level-one and primary sampling units (EAs) as level two. The mixed-effect logistic regression model incorporated fixed effects and cluster-level random effects to account for the within-cluster correlation of clustered data. The two-stage, mixed effect logistic regression model was described by Logit (Yij) = β_0_j + ∑βXi + ϒZj + εj, where β_0_j = β_0 +_ μj, μj ∼ N (0, σ^2^_u_) and εj = ε_0_ + εj, εj ∼ N (0, σ^2^ ε) (28). In this model, logit (Yij) = ln (Yij/(1-Yij)) was the log-odds for IACFP, also known as “the logit link. The symbol “Yij” was a probability of IACFP for a mother of IYC l “i” in any EA, rural or urban region, “j.” “β_0_j” was the cluster random intercept. “εj” was the residual for each cluster “j.” “β” was the fixed effect regression coefficient, “Xi” were level-1 predictors, and “ϒZj” were level-2 factors in cluster j.

We selected four models for multilevel analysis: Model I was an empty model, which had no individual or community-level variables; Model II was adjusted for individual-level variables; Model III was adjusted for community-level variables; and Model IV was adjusted for both the individual and community-level variables. Model comparison was done using deviance, and the model with the smallest value of deviance was selected as the final best-fit model. Adjusted odds ratios (AOR) with their corresponding 95% CI were calculated to identify the determinants of IACFP with a *p*-value of less than 0.05.

In the random-effects model, we computed the intra-class correlation coefficient (ICC), median odds ratio (MOR), and proportional change in variance (PCV) for measures of variation between clusters. The fixed effect model had only one source of variability (εj, with its variance σ^2^μ), while the random effect model had two components of variability (εj and ε_0_ with variances σ^2^μ and σ^2^ε respectively). These two sources of variability showed the variability between predictors that were in the same group, measured by the within-group variance σ^2^μ, and the variability between observations that were in different groups, measured by the between-group variance σ^2^ε. The proportion of between-group variance (σ^2^ε) to the total variance (σ^2^μ + σ^2^ε) is called the intraclass correlation (29). In the random-effects model, we computed the intra-class correlation coefficient (ICC), median odds ratio (MOR), and proportional change in variance (PCV) for measures of variation between clusters. The fixed effect model had only one source of variability (εj with its variance σ^2^μ), while the random effect model had two components of variability (εj and ε_0_ with variances σ^2^μ and σ^2^ε respectively). These two sources of variability showed the variability between predictors that were in the same group, measured by the within-group variance σ^2^μ, and the variability between observations that were in different groups, measured by the between-group variance σ^2^ε. The proportion of between-group variance (σ^2^ε) to the total variance (σ^2^μ + σ^2^ε) is called the intraclass correlation (29). To calculate ICC we used the formula: ICC (ρ) = $\frac{\sigma^{2}\varepsilon }{\sigma^{2}\varepsilon +\sigma^{2}\mu }\text{;}$ σ^2^μ = $\frac{\pi^{2}}{3}$ = 3.29 was within-group variance (σ^2^μ) (30); σ^2^μ = $\frac{\pi^{2}}{3}$ = 3.29 was within-group variance (σ^2^μ). The ICC quantified the variation of IACFP within clusters. The ICC ranges from 0 to 1, and ICC = 0 means perfect independence of residuals, or the observations do not depend on clusters. In contrast, ICC = 1 or lower indicates residual dependencies, i.e., there is a variation of observations between clusters (30).

Similarly, MOR described the cluster heterogeneity obtained by comparing two mothers of IYC with IACFPs from two different clusters that were chosen at random. The MOR is defined as the median value of the odds ratio between the highest and lowest risk areas when comparing two individuals from two different randomly selected clusters. It was calculated using the following formula: MOR = exp $\sqrt{2}x\;VA2\;x\;0.6745$ = exp (0.95xVA) (30). Here, VA was the estimated variance of clusters. The MOR is always greater than or equal to 1. If the MOR is 1, there is no variation between clusters. The total variation attributed to individual and cluster-level factors in each model was measured by the proportional change in variance (PCV), which was computed by PCV = $\frac{VA-VB}{VA}\;X\;100$ (30). The VA was the variance of the initial model, and the VB was the variance of the next model.

## Results

### Sociodemographic and socioeconomic characteristics of participants

In this study, we used a weighted sample of 1,463 infants and young children aged 6 to 23 months who were living with their mother or caregiver. About half (49.56%) of mothers were from the age group of 25–34 years, and their median age was 27 years (IQR 23–30). The majority (95%) of mothers were married. Six hundred fifty (44.43%) women had no education. Among the respondents, 295 (20.16%) and 309 (21%) were the poorest and the poorer, respectively. One-fourth (24.06%) of mothers had no ANC visit, and nearly half (44.91%) of women gave birth in their homes. The majority of newborns, 1,265 (86.47%), had a post-natal check-up within two months after delivery, and the majority of young children 1,246 (85.17%), were still breastfeeding (Table 1).

**Table 1:** Socio-demographic and economic characteristics of respondents in Ethiopia, 2019.

Factors	Categories	Weighted frequency (*n*)	Percent (%)
Respondents age groups:	15–24	473	32.33
	25–34	725	49.56
	35–49	265	18.11
Religion:	Orthodox	539	36.84
	Muslim	474	32.40
	Protestant	413	28.23
	Others	37	2.53
Highest Educational Level of mothers	No Education	650	44.43
	Primary	609	41.63
	Secondary	120	8.20
	Higher	84	5.74
Marital status	never in union	6	0.40
	currently in union	1,394	95.33
	formerly in union	63	4.27
Household has radio	No	1,095	74.85
	Yes	368	25.15
Number of under five children	No child	6	0.41
	1–2 children	1,309	89.47
	> = 3 children	148	10.12
living children and current pregnancy combined	1–2	697	47.64
	> = 3	766	52.36
House hold Wealth index	Poorest	295	20.16
	Poorer	309	21.12
	Middle	276	18.87
	Richer	258	17.64
	Richest	325	22.21
Contraceptive use and intention	No	750	51.26
	Yes	713	48.74
Birth order number	one	354	24.20
	two-four	691	47.23
	five or more	418	28.57
Antenatal visits during pregnancy	No ANC visit	352	24.06
	One visit	62	4.24
	2–3 visits	389	26.59
	4 or more visits	660	45.11
Place of delivery	Home	657	44.91
	Health facility	806	55.09
Baby post-natal check-up within two months	No	1,265	86.47
	Yes	198	13.53
Currently breastfeeding	No	217	14.83
	Yes	1,246	85.17
Age of child in months	6–11	476	32.54
	12–17	552	37.73
	18–23	435	29.73
Child received vitamin A1, (the most recent)	No	723	49.42
	Yes	740	50.58

### Community-level characteristics of study participants

The majority of study participants were in rural areas: 1050 (71.77%) (Table 2), the majority of whom, 958 (91.25%), had IACFPs (Table 3).

**Table 2:** Community-level characteristics of respondents in Ethiopia, 2019.

Factors	Categories	Weighted frequency (n)	Percent (%)
Type of place of residence	Urban	413	28.23
	Rural	1,050	71.77

**Table 3:** The proportions of complementary feeding indicators and the national prevalence of inappropriate complementary feeding practices in Ethiopia, 2019.

Infant and young child feeding practice indicators	Categories	Proportion (%)	95% CI
ISSSF for 6–8 months	Inadequate	16.73	14.90–18.74
	Adequate	83.27	81.26–85.10
MDD 6–23 months	Inadequate	85. 98	84.10–87.67
	Adequate	14. 02	12.33–15. 90
MMF 6–23 months	Inadequate	45.00	42.47–47.57
	Adequate	55.000	52.43–57.53
MAD	Inadequate	88.50	86.76–90.04
	Adequate	11.50	9.96–13.24
Complementary feeding practice	ACFP	9.78	8.36–11.42
	IACFP	90. 22	88.58–91.64

### The proportions of inadequate complementary feeding indicators and the national prevalence of inappropriate complementary feeding practices (IACFP)

In this study, the proportions of total inadequate ISSSF for 6–8 months, MDD for 6–23 months, MMF for 6–23 months, and MAD for 6–23 months were 16.73, 85.98, 45, and 88.5%, respectively. The prevalence of IACFPs among IYC in Ethiopia was 90.22% (95% CI: 88.58–91.64) (Table 3).

The prevalence of IACFPs varied from region to region in Ethiopia. Somalia and Afar regions had the highest prevalence of IACFP (98.79 and 98.30%, respectively), compared with the national prevalence, whereas the Oromia region (84.93%) and Addis Ababa (80.21%) had the lowest compared with others (Table 4).

**Table 4:** Percent distribution of inappropriate complementary feeding practices among infants and young children aged 6–23 months who are living with their mother by region in Ethiopia, EMDHS 2019.

Sr. No	Region	Complementary feeding practices	
		Appropriate (%)	Inappropriate (%)
Region	Tigray	7.58	92.42
	Afar	1.70	98.30
	Amhara	5.24	94.76
	Oromia	15.07	84.93
	Somali	1.21	98.79
	Ben. Gumuz	7.68	92.32
	SNNPR	7.50	92.50
	Gambela	8.19	91.81
	Harari	10.73	89.27
	Addis Ababa	19.79	80.21
	Dire Dawa	10.69	89.31
	National	9.78	90.22
Type of place of residence	Urban	12.41	87.59	Rural	8.75	91.25

## Spatial variation of inappropriate complementary feeding practices in Ethiopia

### Global spatial autocorrelation (Moran’s *I*) analysis

**Figure 1:**
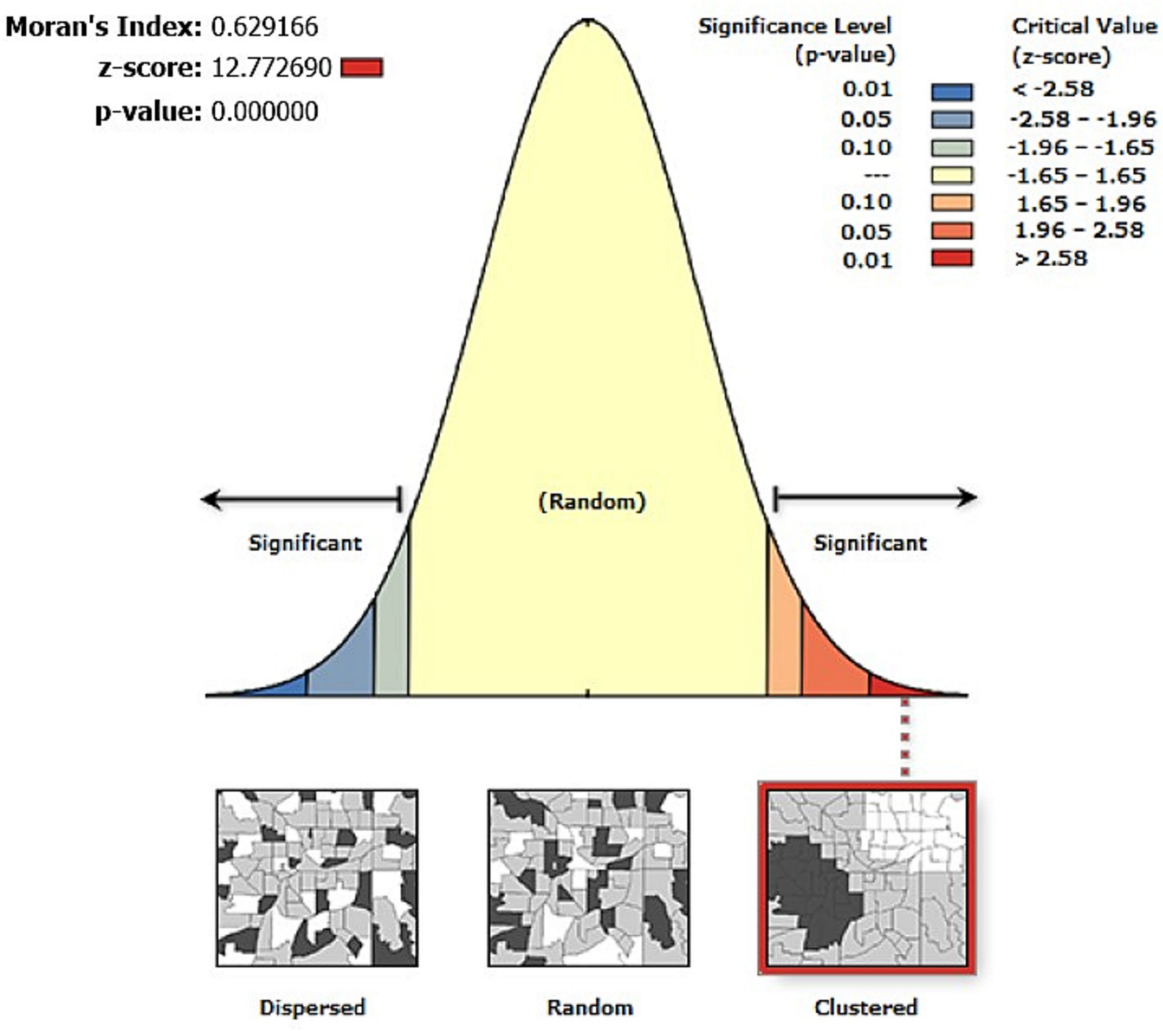
Spatial patterns of IACFP among mothers of IYC in Ethiopia, 2019.

The spatial patterns of IACFPs among mothers of IYC in Ethiopia were clustered. The global spatial autocorrelation analysis showed that there were significant clustered patterns of IACFPs in the regions of Ethiopia (Global Moran’s *I = 0.629,* Z-score = 12.773, value of *p* <0.001). This stated that IACFPs in IYC with similar patterns were interdependent. Global Moran’s output showed that the Z-score was high and positive, with a highly significant value of p that would be interpreted as 99% confidence for the clustering of IACFPs across regions in Ethiopia. The figures below showed the clustered patterns (on the right side) with high rates of IACFPs across regions in Ethiopia. The bright red and blue colors (to the right and left sides) indicated an increased significance level for which the likelihood of clustered patterns occurring by random chance was less than 1% (Figure 1).

### Hotspots (Getis-Ord Gi*) analysis of IACFPs in Ethiopia, 2019

**Figure 2:**
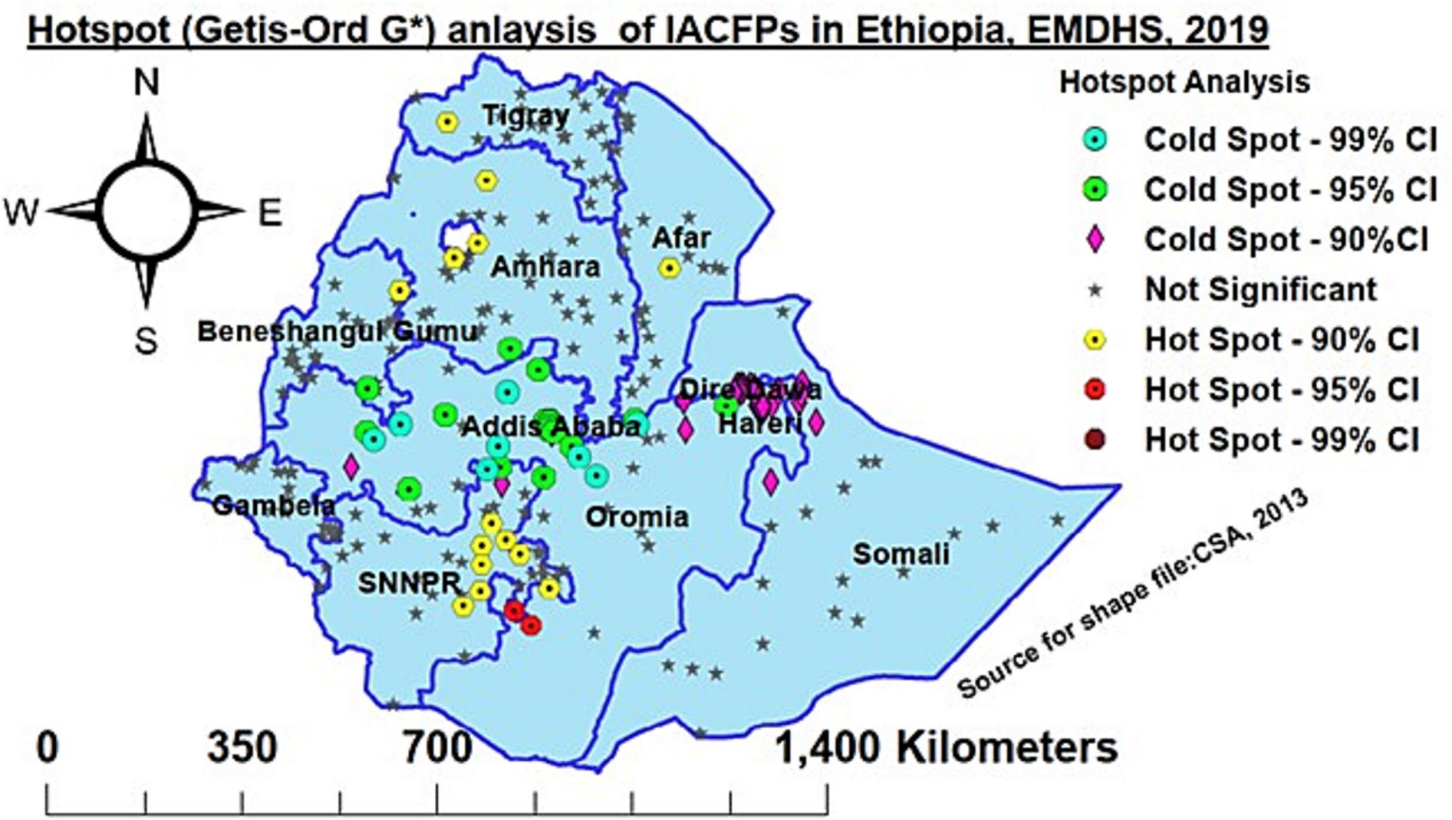
Hotspot analysis of IACFP among mothers of IYC aged 6–23 months in Ethiopia, 2019.

The regions of western Tigray, Afar, western Amhara, and Southern Oromia, as well as eastern SNNPR, were hotspot areas of IACFPs, whereas Addis Ababa, central Oromia, the southern Amhara region, northern SNNPR, southern Benishangul Gumuz, Harari, Dre-Dawa, and the Northern Somali region were cold spot areas (Figure 2).

### Analysis of the inappropriate complementary feeding practice cluster and outlier (Anselin local Moran’s I)

**Figure 3:**
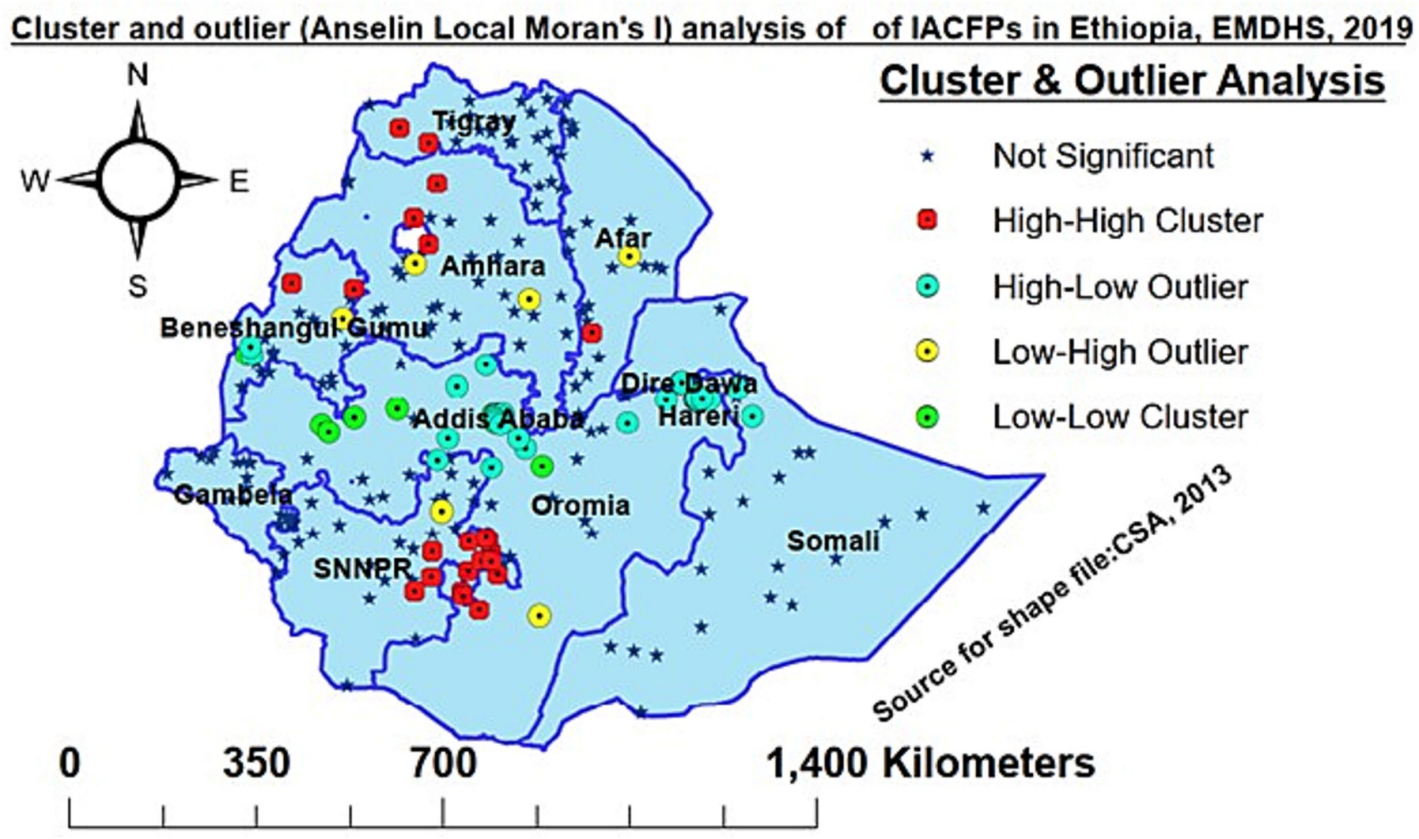
Cluster and outlier (Anselin Local Moran’s I) analysis of IACFP among mothers of IYC in Ethiopia, 2019.

In this study, the high-high significant cluster areas were observed in the regions of western Tigray, southern Afar, northwestern Amhara, southern Oromia, northern Benishangul Gumuz, and eastern SNNPR regions, whereas the low-low significant clusters were observed in central, western, and eastern Oromia, southwestern Benishangul Gumuz, Addis Ababa, Harari, Dre-Dawa, and Jigjiga in Somali regions. The low-high outliers were detected in Afar, Amhara, southern Oromia, and northern SNNPR, while Addis Ababa, Dire-Dawa, Harari, the central and eastern Oromia region, northern SNNPR, Southern Benishangul Gumuz, and Jigjiga Somali were observed as high-low outliers. The High-High cluster stated that the high prevalence of IACFPs was surrounded by high rates; High-Low means the high prevalence of IACFPs was surrounded by low rates; and Low-High means the low prevalence of IACFPs was surrounded by high rates. Low-low revealed a low prevalence of IACFPs surrounded by low rates (Figure 3).

### Spatial interpolation of IACFP among mothers of IYC in Ethiopia

**Figure 4:**
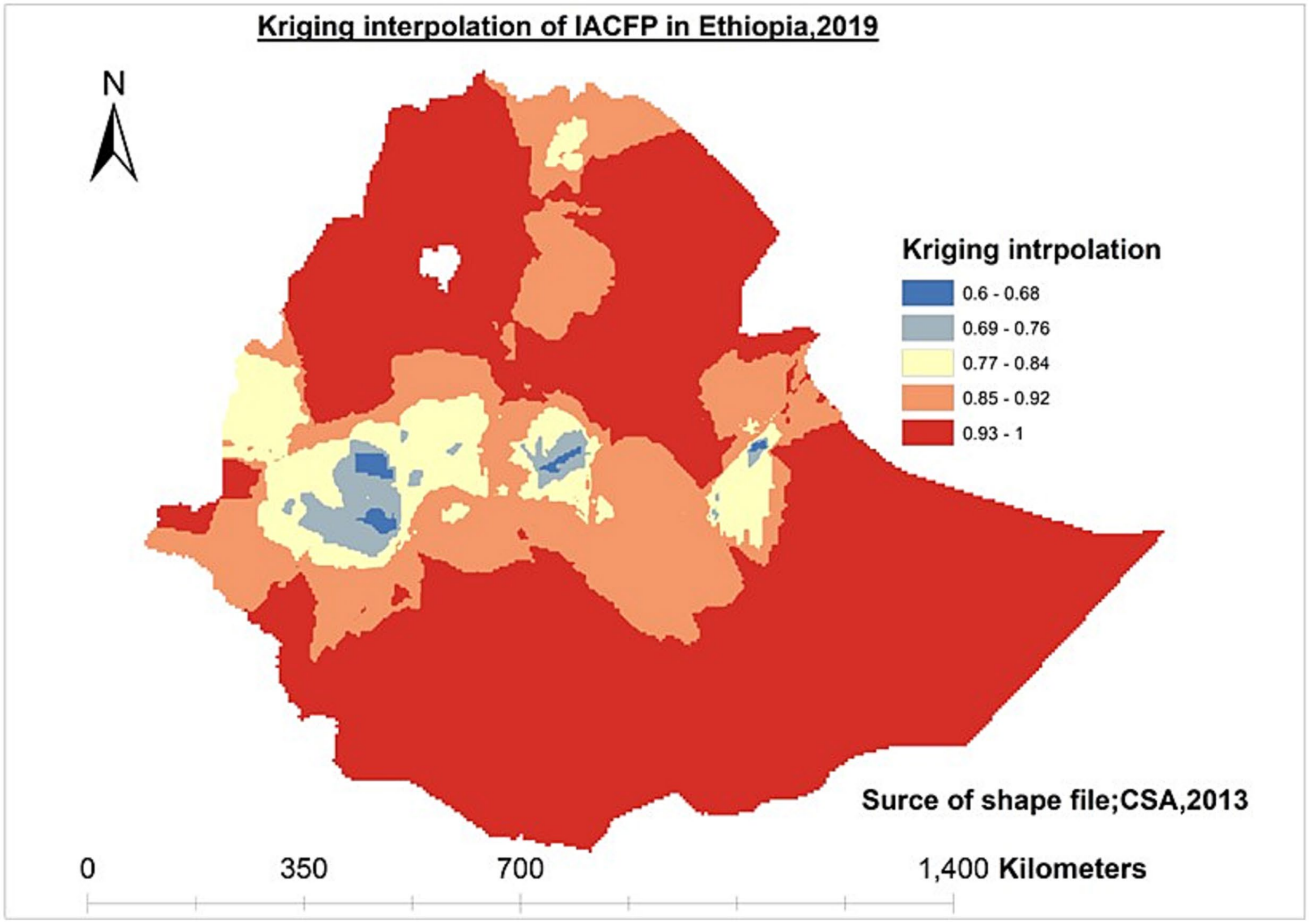
Ordinary kriging interpolation of IACFP among IYC in Ethiopia, 2019.

According to this study, Tigray, Afar, Amhara, some regions of Oromia, the Somali region, Benishangul Gumuz region, SNNPR, and Gambela were predicted to have a high prevalence of IACFPs in the unobserved enumeration areas, whereas Addis Abeba, the western Oromia region, Dire-Dawa, and Harari regions were predicted to have a low prevalence (Figure 4).

### SaTScan cluster analysis of IACFP among mothers of IYC in Ethiopia, 2019

In the five most likely clusters, a total of 92 significant clusters were found. The primary significant big cluster of spatial windows encompassed mainly the western Oromia region (West Shewa zone, southwest Shewa zone, West Welega zone, East Welega zone, Kelam Welega zone, Jima zone, and Illubabor zone); the northern parts of SNNPR (Adiya, Keffa zone); southern parts of Amhara region (East Gojjam (Yejube and D/Eliyas), West Gojjam (Burie and Shindi), and Awi zone (Azena and Ambala); and southern Benishangul Gumuz region. The primary window was located at 9.012845 N, 36.354912 E, /207.88 km radius. In the primary SaTScan window, clusters had 11% less likely to have IACFPs compared with those outside the window (RR = 0.89, LLR = 30.89, *p*-value <0.001).

The second most likely SaTScan window covered Southeast Oromia (West Arsi and Guji Bale) and the eastern parts of SNNPR (Sidama and Gedeo zones), which were located at 6.672214 N, 38.974495 E, /115.31 km radius. Clusters in the second window were 1.13 times more likely to have IACFP compared with those outside the window (RR = 1.13, LLR = 21.44, *p*-value <0.001).

The third most likely SaTScan cluster encompassed the East Shewa and West Hararghe zones of the Oromia region, and it was located at 8.313592 N, 40.103390 E/86.84 km radius. Clusters in this window were 24% lower for having IACFPs than those outside the window (RR = 0.76, LLR = 12.37, *p*-value less than 0.01).

The fourth significant and most likely SaTScan window included parts of Ethiopia’s Northwestern and Northern zones (the central Gondar zone, the North Gondar zone, and the West Gondar zone; Kafta Humera; Wilkeite Tegedie; Maytsebri; Shire; Aksum). This window was found at 13.881625 N, 37.111279 E/196.05 km radius, and the clusters in this window were 1.12 times higher than those outside this window for having IACFPs (RR = 1.12, LLR = 12.22, *p*-value less than 0.01).

**Figure 5:**
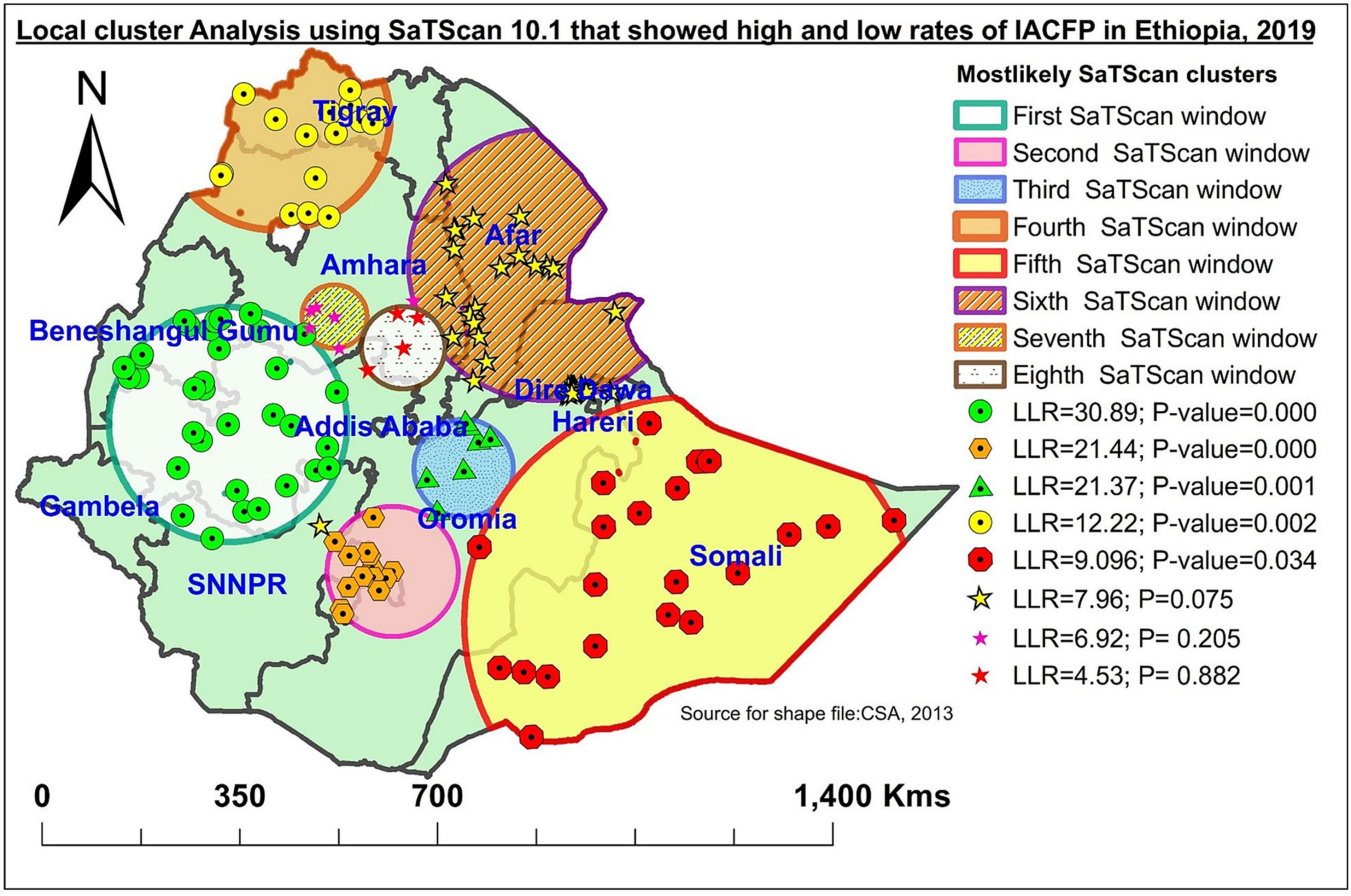
SaTScan cluster analysis of IACFPs among mothers of IYC in Ethiopia, 2019.

The fifth most likely significant SaTScan window was found in the eastern and southeastern parts of Ethiopia, which encompassed mainly the Ethio-Somali region (Degehabur, Gode) and the eastern Oromia region. The clusters in the fifth window were 1.12 times higher than those outside this window for having IACFPs (RR = 1.12, LLR = 9.10, *p*-value less than 0.05), and they were located at 5.856584 N, 43.726017 E/399.27 km radius (Table 5 and Figure 5).

**Table 5:** Significant SaTScan cluster analysis IACFPs among mothers of IYC in Ethiopia, 2019.

Cluster	Significant EAs (clusters) detected	Coordinates/Radius	Population	Cases	RR	LLR	*p*-value
Primary	93, 120, 92, 168, 98, 167, 169, 87, 94, 97, 119, 164, 95, 96, 91, 166, 174, 77, 112, 86, 194, 148, 163, 161, 155, 156, 171, 150, 99, 158, 80, 195, 154, 52	(9.012845 N, 36.354912 E) / 207.88 km	255	192	0.81	30.89	0.000
Secondary	117, 183, 181, 186, 113, 185, 182, 187, 184, 115, 116, 172, 178, 188	(6.672214 N, 38.974495 E) / 115.31 km	192	192	1.13	21.44	0.000
Tertiary	102, 105, 104, 88, 28, 41, 103	(8.313592 N, 40.103390 E) / 86.84 km	68	47	0.76	12.37	<0.01
Fourth	21, 22, 4, 8, 9, 56, 1, 55, 85, 6, 7, 13, 84, 82, 12, 83	(13.881625 N, 37.111279 E) / 196.05 km	113	113	1.12	12.22	<0.01
Fifth	137, 138, 123, 135, 142, 136, 145, 134, 140, 131, 141, 122, 132, 133, 124, 125, 143, 144, 129, 111, 139	(5.856584 N, 43.726017 E) / 399.27 km	85	85	1.12	9.10	<0.05

## Multilevel mixed-effect logistic regression analysis

### Model comparison and cluster variation

Model comparison was done by using deviance. Model IV, or the full model, with the smallest value of deviance (629.316), was taken as the best-fitting model (Table 6).

**Table 6:** Model comparison and model fitness.

Model	Deviance
I	813.412
II	646.669
III	714.481
IV	629.316*

*Best fitted model: Deviance for 4th model was the best fitted model (the smallest one).

The ICC value for the null model was 50.00% (95% CI: 37.10–62.90%), which informed us to choose multilevel mixed effect logistic regression analysis over the basic model. The null model showed that inappropriate complementary feeding practice (IACFP) was clustered across the communities among mothers of infants and young children in Ethiopia (VA^2^ = 3.29; *p* value less than 0.001). An intercept-only model revealed that 50.00% of the variation in the odds of IACFP could be attributed to community-level factors based on the output of the ICC value. The full model, after adjusting the individual and community-level factors, revealed that a 12.30% variation of IACFP across the communities was observed (*p*-value = 0.05 (Table 7).

**Table 7:** Measurement for variation of inappropriate complementary feeding practices at the cluster level to perform mixed-effect binary logistic regression analysis.

Measure of variation	Model – I^a^	*p*-value	Model-II^b^	*p*-value	Model-III^c^	*p*-value	Model- IV^d^	*p*-value
Community level Variance (SE)	3.2894 (0.8860)	<0.001	0.5534 (0.2416)	<0.05	1.2553 (0.2224)	<0.001	0.4614 (0.2534)	0.05
ICC %	50.00		14.40		27.62		12.30	
PCV %	Ref.		83.176		53.867		63.244	
MOR	22.758		1.692		3.295		1.550	
Model fit statistics for best fitted model								
Deviance	813.412		646.669		714.481		629.316*	

SE, standard error; ICC, intraclass correlation; PCV, Proportional Change in Variance, expresses the change in the cluster level variance between the null model and the individual level model, and between the individual level model and the model further including the community level covariates; MOR = Median Odds Ratio; Deviance = (−2LLR).

Model Ia - Empty model without any explanatory variable.

Model IIb - Adjusted for individual-level factors.

Model IIIc - Adjusted for community-level factors.

Model IVd - Full model adjusted for both individual and community-level factors.

The MOR also showed that IACFP among mothers of infants and young children was attributed to community-level factors. In the intercept-only model, the MOR value was 22.76; this indicated that there was variation (clustering) between communities, and its value was decreased to 1.55 in model IV when we added all variables that indicated the community-level variations of inappropriate complementary feeding practices (Table 7).

An overall variation predicted for individual and cluster-level factors in each model was measured by the proportional change in variance (PCV), which was computed as 83.18, 53.87, and 63.24% in models II, III, and IV, respectively. This showed that there was clustering within communities, which informed us to use mixed-effect logistic regression (Table 7).

### Factors associated with IACFP among mothers of IYC aged 6–23 months in Ethiopia

Individual and community-level factors were selected at the 0.2 level of significance in bivariable analysis. In multivariable multilevel mixed-effects logistic regression analysis, the educational status of mothers, availability of radio in the household, household wealth index, babies’ post-natal check-up within two months, breast-feeding status, a child receiving vitamin A1, and child age were significantly associated with inappropriate complementary feeding practices (Table 8).

**Table 8:** Factors associated with IACFPs among mothers of IYC aged 6–23 months in Ethiopia, 2019 using mixed-effects binary logistic regression analysis.

Variables	Categories	IACFPs %		Model- I Null model	Model-II: Level –I AOR, (95%CI)	Model-III: Level-2 AOR, (95%CI)	Model-IV: L1 & L2 AOR, (95%CI)
		No	Yes				
Educational Level of mothers	No Education	6.15	93.85		4.23, (1.78–10.08) **		3 0.97, (1.64–9.60) **
	Primary	12.13	87.87		1.64, (0.83–3.22)		1.75, (0.88–3.48)
	Secondary	17.17	82.83		1.45, (0.67–3.12)	,	1.52, (0.70–3.30)
	Higher	10.29	89.71		1.00		1.00
Availability of radio in the household	No	8.21	91.79		1.00		1.00
	Yes	14.46	85.54		0.60, (0.38–0.95) *		0.61, (0.38–0.97) *
Household wealth index status	Poorest	1.94	98.06		6.54, (1.95–22.01) **		4.80, (1.23–18.71*)
	Poorer	7.78	92.22		1.66, (0.75–3.65)		1.73, (0.64–4.66)
	middle	11.94	88.06		1.07, (0.54–2.12)		1.05, (0.42–2.62)
	richer	11.83	88.17		1.46, (0.74–2.90)		1.49, (0.62–3.59)
	Richest	15.35	84.65		1.00		1.00
Living children plus current pregnancy	1–2	12.34	87.66		1.00		1.00
	≥3	7.46	92.54		0.92, (0.49–1.73)		0.87, (0.46–1.66)
Birth order	One	12.29	87.71		1.00		1.00
	Two to four	10.36	89.64		1.43, (0.80–2.56)		1.47, (0.81–2.65)
	five or more	6.71	93.29		1.68, (0.65–4.32)		1.79, (0.69–4.69)
Contraceptive use	No	8.16	91.84		1.24, (0.77–1.99)		1.21, (0.75–1.95)
	Yes	11.49	88.51		1.00		1.00
ANC visit	No ANC visit	9.71	90.29		0.77, (0.34–1.74)		0.87, (0.38–2.01)
	One visit	4.81	95.19		1.39, (0.28–7.00)		1.40, (0.27–7.36)
	2–3 visits	7.99	92.01		1.64, (0.95–2.84)		1.59, (0.90–2.79)
	+4^th^ visit	11.35	88.65		1.00		1.00
Place of delivery	Home	7.61	92.39		1.07, (0.55–2.08)		1.10, (0.56–2.15)
	Health facility	11.56	88.44		1.00		1.00
Baby post-natal check-up within two months	No	8.37	91.63		2.19, (1.29–3.70) **		2.18; (1.28–3.72) **
	Yes	18.79	81.21		1.00		1.00
Currently breast feed	No	4.85	95.15		2.92, (1.36–6.24) **		2.80, (1.29–6.10) **
	Yes	10.64	89.36		1.00		1.00
Child received VA 1	No	4.90	95.10		1.00		1.00
	Yes	14.55	85.45		0.518, (0.31–0.87) *		0.51; (0.30–0.87*
Age months	6–11	6.13	93.87		1.00		1.00
	12–17	12.39	87.61		0.509, (0.29–0.89) *		0.5045; (0.29–0.89) *
	18–23	10.47	89.53		0.580, (0.32–1.05)		0.60, (0.33–1.10)
Region	Tigray	7.58	92.42			1.14;(0.33–3.97)	1.19, (0.39–3.62)
	Afar	1.70	98.30			12.07; (2.00–72.80) **	2.29, (0.41–12.64)
	Amhara	5.24	94.76			2.53;(0.69–9.25)	1.54, (0.47–5.09)
	Oromia	15.07	84.93			0.516;(0.17–1.56)	0.37, (0.14–1.03)
	Somali	1.21	98.79			20.01; (2.07–193.78) *	3.57, (0.34–37.84)
	Benishangul	7.68	92.32			2.39;(0.62–9.25)	1.45, (0.43–4.93)
	SNNPR	7.50	92.50			1.78;(0.52–6.09)	1.31, (0.43–4.00)
	Gambela	8.19	91.81			1.46;(0.41–5.2.00)	0.97, (0.30–3.14)
	Harari	10.73	89.27			1.27; (0.41–3.96)	0.92, (0.33–2.54)
	Addis Ababa	19.79	80.21			1.00	1.00
	Dire-Dawa	10.69	89.31			1.88; (0.59–6.04)	1.38, (0.49–3.91)
Place of residence	Urban	12.41	87.59			1.00	1.00
	Rural	8.75	91.25			3.41;(1.71–6.78) ***	1.15, (0.53–2.51)

* = *p*-value 0.05 ** = *p*-value < 0.01 *** = *p*-value < 001.

The odds of having IACFPs among mothers with no education were 3.97 times (AOR = 3.97; 95% CI: 1.64–9.60) more likely than those with higher education. The likelihood of IACFPs among households that own radio was 39% lower (AOR = 0.61; 95% CI: 0.38–0.97) than that of their counterparts. Households with the lowest wealth index were 4.80 times more likely to have IACFPs than the richest households (AOR = 4.80; 95% CI: 1.23–18.71). The odds of having IACFPs were 2.18 times (AOR = 2.18; 95% CI: 1.28–3.72) higher among mothers of babies who did not have post-natal checkups within two months. Non-breastfed infants and young children were 2.8 times (AOR = 2.80; 95% CI: 1.29–6.10) more likely to have IACFP when compared with breastfed ones. Infants and young children who received vitamin A1 were 49% less likely (AOR = 0.50; 95% CI: 0.30–0.87) compared with those who did not receive vitamin A1. Similarly, compared to 6–11-month-old infants, 12–17-month-old children were 49.55 percent less likely (AOR = 0.5045; 95% CI: 0.29–0.89) to have IACF (Table 8).

### Inappropriate complementary feeding practices had an effect on the nutrition status of IYC aged 6–23 months in Ethiopia in 2019

The prevalence of stunting, under-weight, and wasting in this study was 31.5% (95% CI: 29.13–33.91), 19.1% (95% CI: 17.15–21.20), and 7.9% (95% CI: 6.61–9.340), respectively.

Infants who were not initiated on solid, semi-solid, or soft foods (ISSSF) were 1.96 times (OR = 1.91, 95% CI: 1.23–2.96) and 1.82 times (OR = 1.82, 95% CI: 1.30–2.56) more likely to develop wasting and underweight, respectively, compared with those who were taking ISSSF. Likewise, IYC who did not receive minimum dietary diversity (MDD) were 2.31 times (OR = 2.31, 95% CI: 1.08–4.94), 1.65 times (OR = 1.65, 95% CI: 1.02–2.68), and 1.51 times (OR = 1.51, 95% CI: 1.01–2.25) more likely than their peers to develop wasting, underweight, and stunting. The odds of underweight and stunting among infants and young children who were not receiving the minimum acceptable diet (MAD) were 2.28 times (OR = 2.28, 95% CI: 1.43–3.64) and 1.96 times (OR = 1.96, 95% CI: 1.35–2.84), respectively, when compared with their counterparts. Similarly, IYC with IACFPs were 1.93-fold (OR = 1.93, 95% CI: 1.03–3.62) more likely to be underweight than those with ACFP. The odds of having underweight and stunting among younger children aged18 to 23 months were twofold (OR = 2.10, 95% CI: 1.46–3.00) and three times more likely (OR = 2.96, 95% CI: 2.15–4.08) when compared with 6 to 11 months old (Table 9).

**Table 9:** The effect of IACFPs on the nutrition status of IYC aged 6–23 months, in Ethiopia, 2019.

Sr. No.	Complimentary feeding practice indicators	Categories	Nutritional status		
			Wasting	Underweight	Stunting
			OR, (95% CI)	OR, (95% CI)	OR, (95% CI)
1.	ISSSF 6–8 months	No	1.91, (1.23–2.96) **	1.82, (1.30–2.56) **	1.28, (0.93–1.76)
		Yes	1.00	1.00	1.00
2.	MDD 6–23 months	No	2.31, (1.08–4.94) *	1.65, (1.02–2.68) *	1.51, (1.01–2.25) *
		Yes	1.00	1.00	1.00
3.	MMF 6–23 months	No	1.10, (0.75–1.62)	0.87, (0.65–1.16)	091, (0.71–1.17)
		Yes	1.00	1.00	1.00
4.	MAD 6–23 months	No	1.31, (0.75–2.30)	2.28, (1.43–3.64) **	1.96, (1.35–2.84) ***
		Yes	1.00	1.00	1.00
	Age in months	6–11	1.00	1.00	1.00
		12–17	1.12, (0.72–1.76)	1.29, (0.91–1.834	1.68, (1.23–2.28) **
		18–23	1.14, (0.70–1.84)	2.10, (1.46–3.00) ***	2.96, (2.15–4.08) ***

## Discussion

According to the WHO recommendations, infants and young children (IYC) aged 6 to 23 months need appropriate complementary feeding practices in order to have adequate energy, protein, and micronutrients in addition to breastfeeding for the requirement of optimal growth and development (2). Despite this evidence, the prevalence of inappropriate complementary feeding practices (IACFPs) among IYC in Ethiopia is very high. Therefore, the aim of this study was to explore the spatial patterns and determinants of IACFP as well as its effect on the undernutrition of IYC aged 6–23 months in Ethiopia.

The prevalence of IACFPs among mothers of IYC in Ethiopia in 2020 was 90.22% (95% CI: 87.64–92.48%). This was because the proportions of inadequate ISSSF, MDD, MMF, and MAD in Ethiopia were very high (16.73, 85.98, 45, and 88.5%, respectively) (11). The proportion of inadequate MAD in this study was consistent with that in Eastern Africa (88.44%) (9), SSA (90.11%) (8), and Pakistan (88%) (31), but it was higher than that in Ghana (70.1%) (32), Nepal (70%) (33), and Myanmar (84%) (34). This study, however, discovered higher rates of inadequate MDD than in the DRC (67%) (35), Ghana (48.6%) (32), Gambia (26%) (36), Myanmar (75%) (34), and Pakistan (21%) (31). Similarly, it was also higher in terms of inadequate MMF from Gambia (20%) (36) and Myanmar (42%) (34), but it was consistent with Afghanistan (45%) (37). The variation could be due to the following: the definition of MDD, the use of eight-item foods, variation in sociodemographic and socioeconomic status, cultural beliefs, and sample size variation. The current study was lower with inadequate MMF from Ghana (54%) (32) and Pakistan (38%) (31). Our study was also lower in terms of ISSSF from Gambia (30%) (36) and Ghana (27.4%) (32). This could be due to better awareness creation to begin *CF* based on WHO recommendations by community health extension workers with house-to-house visits in Ethiopia (38).

In this study, IACFP was spatially clustered across regions in Ethiopia. The hotspot areas of IACFP among mothers of IYC were observed in western Tigray, Afar, northwestern Amhara, southern Oromia, and eastern SNNPR. In contrast, Addis Ababa, central Oromia, Southern Amhara, northeastern SNNPR, Southern Benishangul Gumuz, Harari, Dre-Dawa, and northern Somali regions had cold spot areas. Similarly, the prediction of IACFPs among mothers of IYC from unsampled EAs was identified in Tigray, Afar, Amhara, some parts of Oromia, the Somali region, the Beninshangul Gumuz region, SNNPRs, and Gambela regions, while Westen Oromia, western Beninshangul Gumuz, Addis Ababa, Dire-Dawa, and Harari would have lower risks. The variation might be due to the fact that Northwestern, Northeastern, and Southeastern Ethiopia are deserted areas, as well as the seasonal attacks of drought that affect the source of food security and diversity.

In this study, the SaTScan cluster analysis showed high and low rates of IACFP among mothers of IYC in Ethiopia. As a result, the primary and the third most likely major clusters were 19 and 24%, respectively, lower for IACFPs. The primary window encompassed mainly most of the western Oromia region, northern parts of SNNPR, southern parts of Amhara, and the southern Benishangul Gumuz region, and the third window clusters were mainly detected in the eastern Oromia region. This was mainly because these areas are highly productive with diverse crops and fruit and vegetable production that fulfill food security. These areas have also better annual rainfall and irrigation activities that aid in food diversity (39). The second most likely SaTScan window clusters included southwest Oromia and parts of SNNPR which were 1.13 times more likely to have higher rates of IACFPs than areas outside this window. This was consistent with the high prevalence of IACFPs in Sidama (91.6%) (40). The fourth and fifth significant most likely clusters were 1.12 times higher than those outside these windows. The fourth SaTScan window clusters were found primarily in Northwestern and Northern Amhara, and Western Tigray regions, while the fifth most likely SaTScan window was detected in eastern and southeastern parts of Ethiopia that mainly encompassed the Ethio-Somali region. This was because there were low proportions of MDD, MMF, and MAD in these areas (11).

In this study, both individual and community-level factors contributed to the high prevalence of IACFPs. In the best-fit model, both individual and community-level determinants accounted for 63.24% of the variations in IACFPs among mothers of IYC. In the final model, the odds of having IACFP among mothers with no education were 3.97 times more likely than those with more educated mothers. This could be because uneducated women lacked awareness and knowledge, which influenced them to use a variety of foods and frequency of meals, as well as to start complementary feeding for their infants on time. The percentage of MMF among mothers with no education was lower than that among those with secondary education (46% versus 70%) in Ethiopia (11). Educated women had increased use of MDD, MMF, ISSSF, and MAD for their infants and children (9, 35, 41). This was supported by similar studies in Bangladesh (42), India (26), Pakistan (18), and Indonesia (19).

The occurrence of IACFP among households that own radios was 39% lower than that of their counterparts. This could be due to households that listen to the radio gaining good knowledge and awareness about the components of complementary feeding practices. Moreover, listening to the radio may help improve cultural beliefs, which might be one of the barriers to the IACFP (18). This finding was consistent with studies in Ethiopia (43), Gambia (36), and West Africa (44).

This study showed that the poorest households were 4.8 times more likely to have IACFPs than the richest households. This could be because the poorest households would not get a variety of foods (45) and would also not have enough food due to their food insecurity. According to the EMDHS report, the proportion of IYC who met MDD was the lowest (6% in the lowest wealth quintile versus 20% in the highest quintile) (11). This finding was consistent with a study in East Africa (9), West Africa (44), India (26), and Pakistan (18).

In this study, the odds of having IACFPs were 2.18 times higher among mothers of babies who did not have any post-natal checkup within two months than their counterparts. This might be because mothers with no PNC checkups do not get health education and awareness, which would improve the use of diverse foods and increase their infants’ meal frequency (45). Improved health education has been reported to be a promoter of *CF* (18). This was consistent with studies in southern Ethiopia (40, 46), DR. Congo (35), and Ghana (32).

According to this study, infants and young children who were not breastfed had a 2.8 times higher chance of receiving inappropriate complementary foods than those who had been breastfed. This might be due to the fact that those children who were not breastfed had an increased tendency to acquire infection; as a result, they could have had a decreased appetite. Breast milk contains all the nutrients, including immunoglobulin, that help protect against infection, but non-breastfed children lose this advantage (2). This finding was supported by a study in Pakistan (18).

In the current study, infants and young children who received vitamin A1 were 49% less likely to have IACFPs when compared with those who did not receive vitamin A1. This could be attributed to mothers of IYC getting health education and becoming aware of appropriate complementary feeding practices at the time when IYC received vitamin A-1. Moreover, vitamin-A supplementation builds infants’ immunity and protects them from developing different infectious diseases, which might be one reason for decreasing appetite among infants.

This study revealed that 12–17-month-old children were 49.6% less likely to have IACFPs compared with 6-to 11-month-old infants. This might be because young children aged 12–17 months had better access to MDD (45) and MAD (9). They might have an improved appetite because of growth and development. It was consistent with previous studies (16, 47).

The current study found that the prevalence of stunting, underweight, and wasting were 31.47 percent (95% CI: 29.13–33.91%), 19.09 percent (95% CI: 17.15–21.20%), and 7.89 percent (95% CI: 6.61–9.40%) among IYC aged 6–23 months, respectively. Stunting in this study was consistent with studies in Tanzania (31%) (48), Malawi (31.9%) (49), and India (29.1%) (50). However, in our study, underweight was higher compared with studies in Tanzania (14%) (48) and Malawi (9.9%) (49). The prevalence of underweight and wasting in this study was lower than in studies in Indonesia (26 and 23%, respectively) (51) and India (43.4 and 43.7%, respectively) (50). The stunting in this study was lower than in a study in Rawad (39%) (52), but higher than in studies in Indonesia (28%) (51), Mongolia (6.3%) (14), and rural areas of China (7.1%) (53). Wasting in this study was also higher than in studies in Tanzania (6%) (48) and rural areas of China (3.0%) (53). This could be because of better complementary feeding practices, sample sizes, and sociodemographic variations.

Evidence showed that inappropriate feeding of IYC was linked with different levels of undernutrition (54). In this study, infants aged 6–8 months who did not receive timely ISSSF were 1.96 and 1.82 times more likely to develop wasting and underweight, respectively, compared with those taking ISSSF.

Similarly, IYC who were not fed a diverse diet were 2.31, 1.65, and 1.51 times more likely to develop wasting, underweight, and stunting than those who had received a minimum diversity of foods. This finding was consistent with a similar study in Tanzania, which showed that children who did not receive the MDD had a 1.37-and 1.49-fold higher likelihood of being stunted and underweight, respectively, but no difference in wasting (48). Our finding was also supported by another study in Tanzania that reported that children with low MDD were likely to be stunted (5). Consumption of a diverse diet was significantly associated with a reduction in stunting, wasting, and being underweight in IYC (48, 55).

In this study, the proportion of stunting, underweight, and wasting in this study was 31.5, 19.1, and 7.9%, respectively. Infants and young children who did not receive the minimum acceptable dietary standards were 2.28 times and 1.96 times more likely to develop underweight and stunting, respectively.

The findings of this study showed that as age increased, the likelihood of developing undernutrition increased. Younger children (18–23 months) were twice as likely to develop underweight as 6-to 11-month-old infants. Similarly, younger children aged 12–17 months and 18–23 months were 1.68 and 2.96 times, respectively, more likely to develop stunting compared with 6–11-month-old infants. This could be because the demand for nutritional requirements increased as children’s ages increased, but if younger children did not receive the standard requirements on time, they would have become underweight and stunted over time. This was consistent with a study in northern Ethiopia (56) and Rwanda (52).

Strength and limitation of the study: This study is considered to be nationally representative since we used nationally collected EMDHS data, and we conducted a spatial analysis to explore spatial variation, including local cluster analysis with high and low rates of inappropriate complementary feeding that would be very important for designing interventions at the community level. We performed a multilevel mixed-effect model for the cluster-level effect of correlations, which provides a better estimate of the level of association. Moreover, assessing the effect of inappropriate complementary feeding practices on the nutritional status of IYC facilitates decision-making. However, because the data were mini-EDHS, we did not find other important variables, such as the occupation status of mothers and factors related to their husbands.

## Conclusion and recommendation

This study found a high prevalence of IACFP, which has an effect on the undernutrition of YIC. The spatial patterns of IACFPs among mothers of IYC in Ethiopia were clustered across regions. In western Tigray, Afar, western Amhara, southern Oromia, and eastern SNNPR, hotspot areas of IACFP were discovered. The most likely significant clusters with a high and low risk of having IACFP encompass western Ethiopia (low risk), southern Ethiopia (high risk), central Ethiopia (low risk), northwestern Ethiopia (high risk), and eastern and southeastern Ethiopia (high risk).

Mothers with no education, poorest households, mothers who did not have post-natal checkups, non-breastfeed status were positively associated with IACFPs, while the availability of radio in the HH, vitamin A supplementation at 6 months, and child age older than 12 months were negatively associated with IACFP. Infants and young children aged 6–23 months who did not receive adequate ISSF, MDD, or MAD had an increased risk of wasting, being underweight, or being stunted in Ethiopia.

Therefore, the government of Ethiopia and stakeholders should take action in the high-risk areas, especially supporting uneducated mothers and the poorest households, increasing PNC visits and breastfeeding status to scale up appropriate complementary feeding in Ethiopia.

## Data availability statement

The original contributions presented in the study are included in the article/supplementary material, further inquiries can be directed to the corresponding author.

## Author contributions

ND: conceived and designed the study, wrote the original draft and acquired data set, performed data extraction, cleaning, and management, conducted data analysis and interpretation, drafted the article, and did the final editing of the manuscript. FA, DS, MCA, and MAA: designed the study, reviewed the first draft, extracted, cleaned, and managed data, participated in analysis and interpretation of data. All authors contributed to the article and approved the submitted version.

## Figures and Tables

**Table:** 

ANC	Antenatal Care
AOR	Adjusted Odds Ratio
ArcGIS	Geographic Information System
*CF*	complementary feeding
CI	confidence interval
CSA	the Central Statistics Agency
DHS	Demographic Health Survey
EAs	Enumeration Areas
EDHS	Ethiopian Demographic and Health Survey
EMDHS	Ethiopian Mini-Demographic and Health Survey Household
IACFP	inappropriate complementary feeding practices
ICC	intra-class correlation coefficient
IQR	interquartile range
ISSSF	Introduction of solid, semi-solid, or soft foods
IYC	Infant and young child
IYCFP	infant and young child feeding practices
LBW	low birth weight
MDD	Minimum Dietary Diversity
LLR	log-likelihood ratio
LMIC	low-and middle-income countries
MAD	Minimum Acceptable Diet
MMF	Minimum Meal Frequency
MOR	Median Odds Ratio
NBFC	none breastfed children
OR	odds ratio
PCV	proportional change in variance
RR	relative risk
SNNPRP	South Nations Nationalities and People’s Representatives
SSA	Sub-Saharan Africa
TB	Tuberculosis
VA	variance of the initial model
VB	variance of the model with more terms
WHO	World Health Organization
